# Influences of Aged Bone Marrow Macrophages on Skeletal Health and Senescence

**DOI:** 10.1007/s11914-023-00820-8

**Published:** 2023-09-09

**Authors:** Moritz Pappert, Sundeep Khosla, Madison Doolittle

**Affiliations:** 1 Division of Endocrinology, Diabetes and Metabolism, Mayo Clinic, Rochester, MN USA; 2 Robert and Arlene Kogod Center On Aging, Mayo Clinic, Rochester, MN USA; 3 Department of Medicine, Paracelsus Medical University, Salzburg, Austria

**Keywords:** Aging, Immunosenescence, Myeloid, Macrophage, Senescence, Phagocytosis

## Abstract

**Purpose of Review:**

The purpose of this review is to discuss the role of macrophages in the regulation of skeletal health with age, particularly in regard to both established and unexplored mechanisms in driving inflammation and senescence.

**Recent Findings:**

A multitude of research has uncovered mechanisms of intrinsic aging in macrophages, detrimental factors released by these immune cells, and crosstalk from senescent mesenchymal cell types, which altogether drive age-related bone loss. Furthermore, bone marrow macrophages were recently proposed to be responsible for the megakaryocytic shift during aging and overall maintenance of the hematopoietic niche. Studies on extra-skeletal macrophages have shed light on possible conserved mechanisms within bone and highlight the importance of these cells in systemic aging.

**Summary:**

Macrophages are a critically important cell type in maintaining skeletal homeostasis with age. New discoveries in this area are of utmost importance in fully understanding the pathogenesis of osteoporosis in aged individuals.

## Introduction

Osteoporosis is generally considered an age-associated disease and is prevalent in elderly individuals [1]. An estimated 25 million Americans over the age of 50 have osteoporosis with approximately 3 million fragility fractures occurring every year in the USA, leading to an annual economic cost of greater than $18 billion [2]. These osteoporotic fractures lead to a high clinical burden in the elderly, with patients aged 65 years or older exhibiting death rates of up to 36% within 12 months of a hip fracture [3]; this unusually high mortality rate is predicted to be driven by the existence of age-related comorbidities. As individuals in this age-group are expected to reach nearly 20% of the global population by 2050 [4], the need to understand the mechanisms of aging on osteoporosis development is more important than ever.

Macrophages are of utmost importance for the innate immune response [5]. The skeleton has a central role in macrophage physiology across the entire organism, as a large subset of these cells arise from circulating monocytes released from the bone marrow. These infiltrating macrophages surge in response to acute injury, including fracture, and have a multitude of functions that promote tissue repair. In addition, other bone-resident macrophages have demonstrated critical roles in maintaining skeletal homeostasis and have highly influential roles in bone resorption. These beneficial roles can turn detrimental upon the establishment of chronic inflammation in the setting of aging. Though the foundational concept of “inflammaging” has long been established, the mechanisms underlying the pathological roles of macrophages on skeletal health with age have only recently been revealed. In this review, we will discuss recent updates on roles of bone marrow macrophages in skeletal aging and senescence, while also touching on unexplored mechanisms that may be conserved from other extra-skeletal macrophage cell types.

## Age-Related Alterations in Bone Marrow Macrophage Subtypes

Extensive heterogeneity has been observed in bone marrow macrophages, revealing complex subtypes that differ in their origin, function, and plasticity. Within the bone microenvironment, the predominant tissue-resident macrophage-lineage populations include osteoclasts, osteal macrophages (“osteomacs”), and erythroblastic island macrophages [6]. Lineage tracing based on single-cell RNA sequencing data revealed that hematopoietic stem cells as well as yolk sac erythro-myeloid progenitors serve as bone-resident macrophage progenitors [7, 8]. Further differentiation into mature osteoclasts requires CSF1 signaling [9], similar to the differentiation of macrophages, along with NFκB signaling, such as RANKL [10]. In contrast with tissue-resident populations, iinfiltrating macrophages originate from common myeloid progenitors (CMPs) that are released into the blood as monocytes, travel to tissue sites, and then differentiate into macrophages [11]. They are generally divided into M1 (classically activated pro-inflammatory) and M2 (alternatively activated anti-inflammatory) subtypes. Activation via pro-inflammatory stimuli, like bacterial lipopolysaccharides (LPS), or high mobility group box 1 protein (HMGB1), leads to an pro-inflammatory response, causing the M1 macrophage to release a cocktail of pro-inflammatory cytokines and chemokines (e.g., IL-6, TNF-α, CXCL9, CXCL10) [12–14]. M2 polarization is induced through Th2 cytokines, like IL-4 and IL-13 [15], promoting the release of anti-inflammatory cytokines like IL-10 and TGF-β [15]. Furthermore, evidence suggests that pro-inflammatory macrophages are able to mute the anti-inflammatory phenotype [16]. It should be mentioned that this classification of macrophages has been criticized as simply painting the two extremes of the macrophage polarization spectrum and not being applicable in vivo [17]. However, due to its clarity and simplicity, the M1/M2 model has been established as a common working model for macrophages and will therefore also be used in this review.

With age, several differences in macrophages can be observed. During aging, an accumulation of inflammatory factors, as well as an altered inflammatory response can be observed, shown to arise from altered macrophage physiology [18, 19]. This chronic inflammatory state that occurs with aging is also known as “inflammaging.” Although the puzzle behind this phenomenon is far from being solved, a number of mechanisms have been proposed. It has been reported that, upon stimulation with inflammatory factors such as LPS, macrophages express CD38, a NADase, and release it into the environment [20]. Nicotinamide adenine dinucleotide (NAD) is involved in redox reactions but also in other key processes, such as cell signaling and DNA repair [21]. Interestingly, a decrease in intracellular NAD with age has been observed, most likely due to inflammaging-induced upregulation of CD38 [22–24]. The decrease of NAD is associated with numerous age-related morbidities, as well as bone mass loss [25, 26]. CD38 has also been found to be specific for M1-like macrophages [27]. Accordingly, while the overall quantity of macrophages remained the same with age, aged mice exhibited increased expression of genes associated with pro-inflammatory M1-macrophages in fracture calluses, with unique overall transcriptomes relative to young mice [28]. Aged mice treated with a CSF1R inhibitor, thereby inhibiting macrophage recruitment, demonstrated improved fracture healing, whereas no effect was observed in young mice. Along these lines, it was found that M2 macrophages promote bone regeneration in the settings of heterotopic ossification and fracture [29]. This evidence outlines likely mechanisms by which age-related alterations in the makeup of macrophage subtypes disrupt skeletal metabolism.

## Macrophage-Mediated Modulation of Bone Loss and Regeneration

Due to the abundance of myeloid lineage cells in the bone marrow, the crosstalk between macrophages and cells maintaining bone tissue integrity is of vast significance. This relationship has been suggested since the initial use of clodronate, a first-generation bisphosphonate, in osteoporotic patients [30], as this drug is also commonly used in mouse experiments to deplete macrophages [31]. In 2002, it was discovered that macrophages release osteogenic proteins, such as BMP-2, which promote osteoblast differentiation [32]. Since then, numerous functions of macrophages have been revealed in promoting the activities of bone formation [33–35], resorption [36], and fracture healing [37]. Conversely, others have found macrophages inhibit bone formation [28, 38], typically as a result of the release of pro-inflammatory interleukins, interferons, and other cytokines [39]. These contradicting results arise from macrophage diversity and/or plasticity, as different effects on skeletal cells may be cell type- or context-dependent. For instance, infiltrating macrophages with a pro-inflammatory “M1-like” phenotype have been demonstrated to drive inflammation with aging in both bone [28] and other tissues [40], while tissue-resident osteomacs have beneficial roles in regulating bone formation and resorption.

Osteomacs are unique to bone and have been reported to influence skeletal metabolism through a number of mechanisms [36]. There is a current debate about their specific cell surface markers; in mice, CD169 and tartrate-resistant acid phosphatase (TRAP) expressing cells have been characterized as osteomacs, while other groups define them as F4/80 + Cd45 + cells [41, 42]. In previous research, CD68 has been used to show osteomac presence in human bone [43]. The close proximity of osteomacs towards other bone lining cells allows them to regulate bone remodeling. In vitro experiments reported that depletion of macrophage-lineage cells resulted in impaired matrix mineralization and reduced bone forming capacities [43]. Recent in vivo studies in mice depleting CD169 + macrophages confirm these results [41]. Furthermore, it was suggested that the reduced bone forming capacity is not caused by increased osteoclast activity but rather due to the paracrine influence of osteomacs on osteoblasts. The observed loss of osteoblasts after osteomac depletion leads to the conclusion that osteomacs promote osteoblast maintenance either through production of survival factors or other unknown mechanisms [41]. Their association with pathologic bone formation when present, and subsequent reduction of pathologic bone formation when depleted, points out their importance for bone formation [44, 45].

Interestingly, recent studies could provide in vivo evidence of apoptotic osteoblast clearance by macrophages [46]. While osteoblasts can develop into osteocytes or bone lining cells, a larger majority of osteoblasts undergo cell death [47]. Considering the suggested role of osteomacs in bone formation, as well as their role in apoptotic clearance, the question arises how mechanisms of clearance of apoptotic osteoblasts and new bone formation are linked [46]. Bone resorption and subsequent bone loss are also heavily influenced by macrophages. TNF-α and IL-1β, both secreted by macrophages, can drive osteoclast formation both directly as well as indirectly through inducing osteocytic production of RANKL [15, 48]. Osteomacs not only support bone resorption by osteoclasts, but also support clearance of the resorption residues [49]. As macrophages have been observed to secrete matrix-metalloproteinases (MMPs) in inflammatory states [50, 51], it is plausible that this also occurs in bone marrow macrophages and may contribute to age-related bone loss; however, this remains to be tested.

## Crosstalk Between Macrophages and Aged Skeletal Cells—a Role for CSF1

Signals emanating from mesenchymal cells have important roles in macrophage recruitment and function. Macrophage colony-stimulating factor (CSF1/M-CSF), a growth factor that drives differentiation of myeloid lineage cells, has recently been of major focus in the bone field. CSF1 deletion was first implicated in bone when it was discovered that the cause of osteopetrosis in the *op/op* mouse model was due to a loss-of-function mutation in *Csf1* [52, 53], resulting in drastically reduced numbers of macrophages and osteoclasts [54]. Within the bone, osteocytes have been observed to release CSF1, and osteocyte-specific deletion of CSF1 led to increased bone mass and reduced osteoclasts, similar to global deletion [55]. Along with non-autonomous effects on myeloid cells, CSF1 was also observed to maintain osteocyte homeostasis both in vitro and in vivo through the inhibition of intracellular ROS production and apoptosis [55]. In addition to osteocytes, it was recently found — independently by two groups — that marrow-adipogenic lineage precursors (MALPs) are the primary source of CSF1 in the bone microenvironment [9, 56]. MALPs are defined by adipocytic gene expression, particularly *Adipoq*, yet exist as stromal and perivascular cells in the marrow cavity [57]. Both groups found that deletion of CSF1 in MALPs drastically reduced whole-marrow CSF1 expression and led to osteopetrosis and reduced bone marrow macrophage abundance upwards of 50%. Although the latter is commonly interpreted as the underlying reason for reduced osteoclast numbers, the resulting non-autonomous effects of reduced bone marrow macrophages on bone metabolism were not investigated. CSF1 in isolation is used to differentiate monocytes towards macrophages in vitro, requiring additional RANKL supplementation to generate osteoclasts [58]. A similar environment exists in the aging skeletal niche, as Ambrosi et al. found that skeletal stem cells (SSCs) upregulate CSF1 with age, but not RANKL [59]. Interestingly, as detailed further below, CSF1 has been implicated in cellular senescence in aged skeletal tissue, although its mechanism of action in age-related bone loss remains unclear. Thus, it will be important to understand the relevance of macrophage-specific CSF1 signaling in skeletal aging.

## Macrophage Influence on HSC Niche

Hematopoetic stem cells (HSCs) reside within the bone marrow and are responsible for producing all blood and immune cells and maintaining them to adequate numbers. During their lifespan, they can experience various routes: They can become quiescent, apoptotic, self-replicate, differentiate into hematopoietic progenitors (HPCs), or even migrate [60]. They are tightly regulated by so-called “niches,” specific microenviroments within the bone, where various cell types (e.g., HSCs, Osteoblasts) influence lineage development. Among other cell types, macrophages have an influential role in regulating these niches [61].

In vivo depletion of macrophages leads to a loss of osteoblasts and more importantly a loss of function of the niche, shown by HSC and HPC mobilization and migration into the periphery [61]. Furthermore, treatment with G-CSF, used to mobilize HSCs, caused osteomacs to disappear from endosteal surfaces, even before osteoblast depletion occurred [61]. This chain of events was remarkably similar to what happened upon depletion of macrophages, underlining their importance in maintaining the niche [61].

Aged myeloid-biased murine HSCs, marked by CD41 expression, also showed increased expression of von Willebrand factor (vWF) and other megakaryocytic genes, indicating a platelet shift (platelet bias) [62]. This skewing towards the megakaryocytic line could also be observed in humans with age [63]. Experiments have shown that aged niche macrophages were able to induce this HSC platelet bias, even in the presence of young macrophages, questioning the role of macrophages in aging HSC niche maintenance [64]. It is suspected that the shift is caused by several distinct mechanisms. Aged bone marrow macrophages shift towards inflammatory M1 phenotype, thus exhibiting increased production of IL-1β [64]. Additionally, through impaired phagocytosis of neutrophils, a main producer of IL-1β, an accumulation of IL-1β could be observed [64]. IL-1β is known to promote maturation of megakaryocytes and eventually of platelets [65]. It should be mentioned that in settings of global inflammation, a reduction of thrombocytes can be observed; thus, the question arises if the platelet bias in age might be beneficial in the context of inflammaging [66].

Osteomacs also maintain the hematopoietic niche in a manner unique to other macrophages [42]. Mohamad et al. found that, although osteomacs and bone marrow macrophages co-express similar markers (e.g., CD45, F4/80, CD11b), a unique osteomac population defined as CD166 + CSF1R + supported HSC function. This is in contrast with previous studies, as it was shown that CD169 and CD68, traditional markers for osteomacs, were also expressed by bone marrow macrophages. Interestingly, substitution of BMDMs for osteomacs in multicellular co-cultures failed to fully support the HSC niche, suggesting that osteomacs are required for the hematopoietic-enhancing activity of osteoblasts and show a unique response upon megakaryocyte stimulation [42].

As CD166 was identified as critical for maintaining the niche and was also expressed on HSCs, it is likely that these cells have a key regulatory function within the HSC niche [67]. Furthermore, osteomacs have been shown to be required for the hematopoetic-enhancing activity of osteoblasts [42]. Overall, osteal and bone marrow-derived macrophages have been shown to be of utmost importance as regulators within the HSC niche. Nonetheless, the HSC niche and the role of macrophages therein remain uncertain and further research is required to definitively describe their role.

## Macrophages and Cellular Senescence

Cellular senescence is a state of growth-arrest concomitant with release of inflammatory cytokines that drive tissue dysfunction and disease [68]. Senescent cells have been shown to accumulate with age in both mouse and human skeletal tissue [69], and clearance of senescent cells in aged mice can delay age-related bone loss [70]. Several characteristics of senescence have been tightly implicated in the physiology of macrophages, which raises important questions regarding their involvement in senescence-driven states of bone loss.

Many macrophage signaling factors have been established in the senescence-associated secretory phenotype (SASP), which suggests that macrophage recruitment and differentiation are promoted by senescent cells. Although originally identified in senescent fibroblasts [71] and tumor cells [72], these SASP factors were only recently linked to senescence in the skeleton [69, 73]. Recently, Saul et al. established a SASP geneset using RNA-seq data from bone biopsies of two aging human cohorts, which was then validated in mouse single-cell RNA-seq samples. Over a fourth of this SASP panel consists of cytokines and chemokine families (e.g., CC & CXC family chemokines, interleukins) with macrophage modulatory functions. Furthermore, SASP-associated cells were found to be highly enriched for MHC-I signaling. In addition to paracrine signaling, it was recently found that senescent cells directly bind to macrophages through their expression of CD47, which inhibits efferocytosis, thereby disrupting tissue homeostasis [74]. As the presence of senescent cells contributes to age-related bone loss, it is plausible that this deleterious phenotype is at least partially driven by their modulation of macrophage function. This has been well-documented in other disease settings, such as cancer [72, 75–77]. However, the extent to which senescence-driven recruitment, differentiation, or polarization of macrophages is able to drive age-related bone loss remains to be tested.

Along with non-autonomous signaling to macrophages, it has been proposed that macrophages themselves may become senescent with age, thereby driving tissue disease. Senescent macrophages have been studied in the context of cancer, whereby they drive tumor progression through a secretory phenotype, and targeting senescent cells can alleviate this phenotype [78, 79]. This phenotype has only recently been linked to skeletal physiology. A recent study by Li et al. concluded that senescent macrophages and neutrophils secrete grancalcin (GCA), which drives age-related bone loss [80]. Grancalcin was found to be upregulated with age and inhibited signaling of the plexin-b2 receptor on mesenchymal cells, thereby downregulating osteogenesis and impairing bone formation. This study established a signaling axis between aged macrophages and mesenchymal cells in regulating bone loss, despite one issue. Although it was concluded that grancalcin was produced by bone marrow macrophages that were senescent, their single-cell analyses revealed that these cells also demonstrated high *Mki67* expression, suggesting they are proliferative. This suggests that, although these macrophages were highly inflammatory, they may not have been truly senescent. This unfortunately is a well-documented occurrence in macrophages, as they can express senescent markers (e.g., express p16 and stain positive for senescence-associated β-galactosidase), yet this is simply part of a senescence-independent physiological response [81]. Moreover, the inflammatory nature of certain macrophage types overlaps greatly with SASP proteins. The difficulties of differentiating senescence from non-senescence in aged macrophages have been discussed at length [82], and will be a significant hurdle in the study of macrophages in senescence-driven states of bone loss. Regardless of whether or not macrophages truly become senescent, it is clear that their secretory profile can have damaging effects on nearby skeletal cells.

## Conclusions

Macrophages within the bone microenvironment have diverse and important functions in regulating bone mass, in addition to their established roles in innate immunity. Macrophage functions have traditionally been studied within the realm of osteoclast differentiation, yet it has been revealed that these cells impress non-autonomous effects upon numerous mesenchymal cell types within the bone microenvironment. Additionally, aged skeletal cells release factors involved in macrophage recruitment, polarization, and differentiation, which implicate these immune cells in states of aging and senescence.

**Fig. 1:**
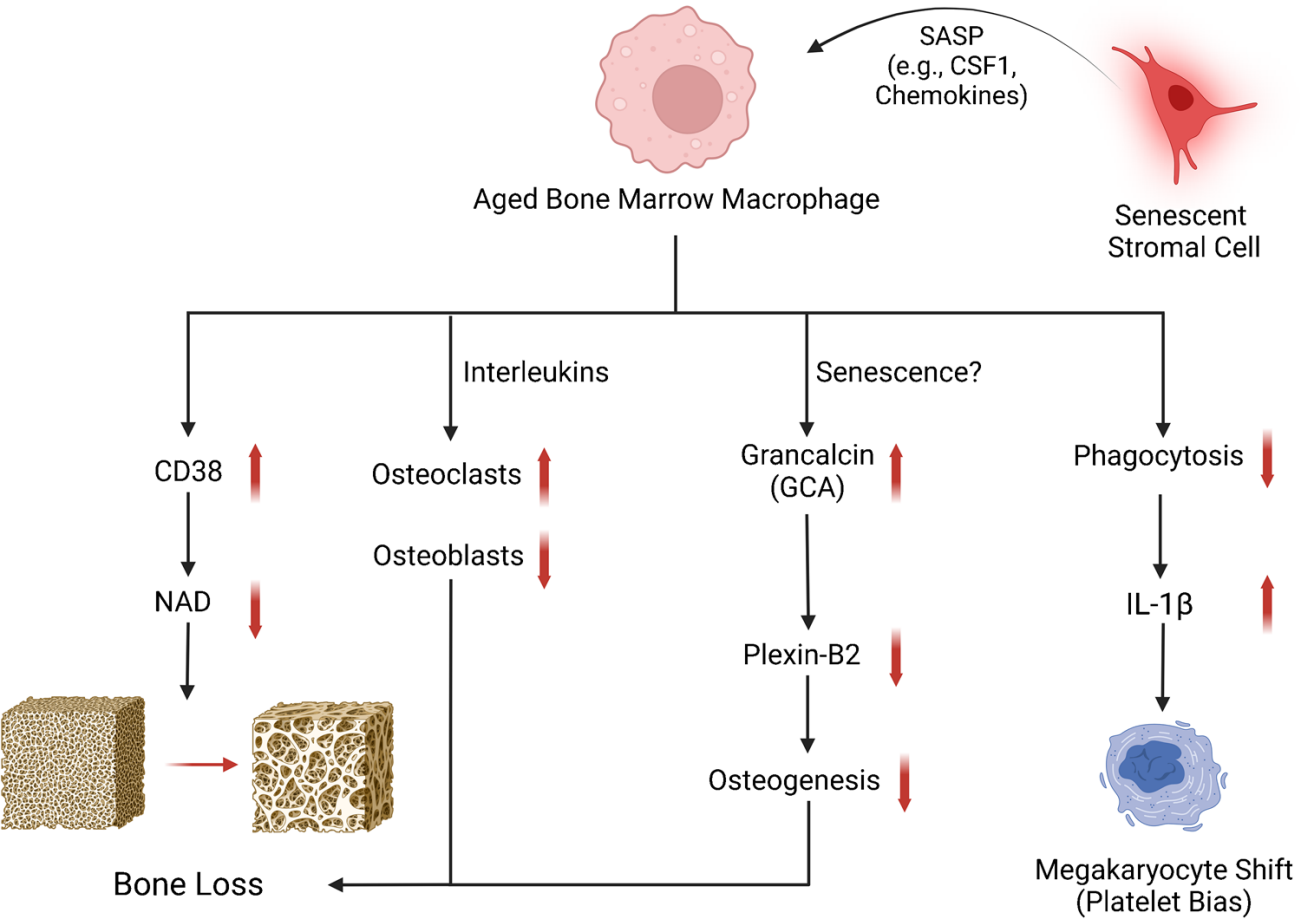
Aged bone marrow macrophages disrupt skeletal metabolism through newly discovered mechanisms. Macrophages have been reported to disrupt the balance of bone formation and resorption with age through intrinsic alterations in macrophage polarization (e.g., CD38 upregulation), promoting osteoclastogenesis, and release of grancalcin upon developing a senescent-like phenotype. Macrophages also disrupt the HSC niche through release of IL-1β and can be influenced by the inflammatory niche generated by aged and senescent skeletal cells. SASP, senescence-associated secretory phenotype. Created with BioRender.com

Although much has been revealed (Fig. 1), several important questions remain. Firstly, although the heterogeneity of infiltrating and tissue-resident macrophage subpopulations in the bone marrow have been classically described, their complexity remains to be elucidated at the single-cell level, as has been done for skeletal muscle macrophages [83, 84]. A major reason this has yet to be accomplished is due to the fragmentation of bone-resident macrophages upon traditional isolation for cytometry [85], which may be circumvented by negative selection techniques or spatially resolved approaches. Secondly, the osteoclast-independent effects of macrophages in driving age-related bone loss require further elucidation, particularly in the setting of CSF1 released by aged SSCs [59]. As CSF1 and other macrophage-related cytokines are additionally released by senescent cells as part of the SASP, establishing a link between the well-documented functions of these cytokines and unexplored age-related cellular mechanisms would be of substantial importance. Thirdly, as described in the previous section, it remains to be observed the extent to which bone marrow macrophages become truly senescent. As many characteristics are shared between senescent cells and terminally differentiated macrophages [82], careful characterization of these immune cells will be required to accurately implicate these cells in senescence-driven disease states.

Although macrophages have been studied for decades, new roles for these cell types are being discovered every year, implicating them in various disease states. As such, the role of these cells in age-related bone loss will be of great interest in fully understanding the pathophysiology of osteoporosis in elderly patients.

## References

[1] Li G, Thabane L, Papaioannou A, Ioannidis G, Levine MA, Adachi JD (2017). An overview of osteoporosis and frailty in the elderly. BMC Musculoskelet Disord.

[2] Office of the Surgeon General (US). Bone Health and Osteoporosis: A Report of the Surgeon General. Rockville (MD): Office of the Surgeon General (US); 2004. Available from: https://www.ncbi.nlm.nih.gov/books/NBK45513/. 20945569

[3] van Staa TP, Dennison EM, Leufkens HG, Cooper C (2001). Epidemiology of fractures in England and Wales. Bone.

[4] Briggs AM, Cross MJ, Hoy DG, Sanchez-Riera L, Blyth FM, Woolf AD (2016). Musculoskeletal health conditions represent a global threat to healthy aging: a report for the 2015 World Health Organization world report on ageing and health. Gerontologist.

[5] Nahrendorf M, Swirski FK (2016). Abandoning M1/M2 for a network model of macrophage function. Circ Res.

[6] Sinder BP, Pettit AR, McCauley LK (2015). Macrophages: their emerging roles in bone. J Bone Miner Res.

[7] Yahara Y, Barrientos T, Tang YJ, Puviindran V, Nadesan P, Zhang H (2020). Erythromyeloid progenitors give rise to a population of osteoclasts that contribute to bone homeostasis and repair. Nat Cell Biol.

[8] Jacome-Galarza CE, Percin GI, Muller JT, Mass E, Lazarov T, Eitler J (2019). Developmental origin, functional maintenance and genetic rescue of osteoclasts. Nature.

[9] Zhong L, Lu J, Fang J, Yao L, Yu W, Gui T, et al. Csf1 from marrow adipogenic precursors is required for osteoclast formation and hematopoiesis in bone. Elife. 2023;12. 10.7554/eLife.82112. 10.7554/eLife.82112 PMC10005765 36779854

[10] Udagawa N, Koide M, Nakamura M, Nakamichi Y, Yamashita T, Uehara S (2021). Osteoclast differentiation by RANKL and OPG signaling pathways. J Bone Miner Metab.

[11] Ginhoux F, Guilliams M (2016). Tissue-resident macrophage ontogeny and homeostasis. Immunity.

[12] Locati M, Curtale G, Mantovani A (2020). Diversity, mechanisms, and significance of macrophage plasticity. Annu Rev Pathol.

[13] Medzhitov R, Janeway C (2000). Innate immune recognition: mechanisms and pathways. Immunol Rev.

[14] Andersson U, Wang H, Palmblad K, Aveberger AC, Bloom O, Erlandsson-Harris H (2000). High mobility group 1 protein (HMG-1) stimulates proinflammatory cytokine synthesis in human monocytes. J Exp Med.

[15] Shapouri-Moghaddam A, Mohammadian S, Vazini H, Taghadosi M, Esmaeili SA, Mardani F (2018). Macrophage plasticity, polarization, and function in health and disease. J Cell Physiol.

[16] Liu G, Ma H, Qiu L, Li L, Cao Y, Ma J (2011). Phenotypic and functional switch of macrophages induced by regulatory CD4+CD25+ T cells in mice. Immunol Cell Biol.

[17] Murray PJ (2017). Macrophage polarization. Annu Rev Physiol.

[18] Singh T, Newman AB (2011). Inflammatory markers in population studies of aging. Ageing Res Rev.

[19] Cunha LL, Perazzio SF, Azzi J, Cravedi P, Riella LV (2020). Remodeling of the immune response with aging: immunosenescence and its potential impact on COVID-19 immune response. Front Immunol.

[20] Lee CU, Song EK, Yoo CH, Kwak YK, Han MK (2012). Lipopolysaccharide induces CD38 expression and solubilization in J774 macrophage cells. Mol Cells.

[21] Chini CCS, Cordeiro HS, Tran NLK, Chini EN. NAD metabolism: role in senescence regulation and aging. Aging Cell. 2023:e13920. 10.1111/acel.13920. 10.1111/acel.13920 PMC10776128 37424179

[22] Zhu XH, Lu M, Lee BY, Ugurbil K, Chen W (2015). In vivo NAD assay reveals the intracellular NAD contents and redox state in healthy human brain and their age dependences. Proc Natl Acad Sci U S A.

[23] Elhassan YS, Kluckova K, Fletcher RS, Schmidt MS, Garten A, Doig CL (2019). Nicotinamide riboside augments the aged human skeletal muscle NAD(+) metabolome and induces transcriptomic and anti-inflammatory signatures. Cell Rep.

[24] Clement J, Wong M, Poljak A, Sachdev P, Braidy N (2019). The plasma NAD(+) metabolome is dysregulated in “normal” aging. Rejuvenation Res.

[25] Chu X, Raju RP (2022). Regulation of NAD(+) metabolism in aging and disease. Metabolism..

[26] Kim HN, Ponte F, Warren A, Ring R, Iyer S, Han L (2021). A decrease in NAD(+) contributes to the loss of osteoprogenitors and bone mass with aging. NPJ Aging Mech Dis.

[27] Jablonski KA, Amici SA, Webb LM, Ruiz-Rosado Jde D, Popovich PG, Partida-Sanchez S (2015). Novel markers to delineate murine M1 and M2 macrophages. PLoS One.

[28] Clark D, Brazina S, Yang F, Hu D, Hsieh CL, Niemi EC (2020). Age-related changes to macrophages are detrimental to fracture healing in mice. Aging Cell.

[29] Olmsted-Davis E, Mejia J, Salisbury E, Gugala Z, Davis AR (2021). A population of M2 macrophages associated with bone formation. Front Immunol.

[30] Kanis JA, McCloskey EV, Beneton MN (1996). Clodronate and osteoporosis. Maturitas.

[31] Michalski MN, Zweifler LE, Sinder BP, Koh AJ, Yamashita J, Roca H (2019). Clodronate-loaded liposome treatment has site-specific skeletal effects. J Dent Res.

[32] Champagne CM, Takebe J, Offenbacher S, Cooper LF (2002). Macrophage cell lines produce osteoinductive signals that include bone morphogenetic protein-2. Bone.

[33] Pirraco RP, Reis RL, Marques AP (2013). Effect of monocytes/macrophages on the early osteogenic differentiation of hBMSCs. J Tissue Eng Regen Med.

[34] Fernandes TJ, Hodge JM, Singh PP, Eeles DG, Collier FM, Holten I (2013). Cord blood-derived macrophage-lineage cells rapidly stimulate osteoblastic maturation in mesenchymal stem cells in a glycoprotein-130 dependent manner. PLoS One.

[35] Nicolaidou V, Wong MM, Redpath AN, Ersek A, Baban DF, Williams LM (2012). Monocytes induce STAT3 activation in human mesenchymal stem cells to promote osteoblast formation. PLoS One.

[36] Yao Y, Cai X, Ren F, Ye Y, Wang F, Zheng C (2021). The macrophage-osteoclast axis in osteoimmunity and osteo-related diseases. Front Immunol.

[37] Guihard P, Boutet MA, Brounais-Le Royer B, Gamblin AL, Amiaud J, Renaud A (2015). Oncostatin m, an inflammatory cytokine produced by macrophages, supports intramembranous bone healing in a mouse model of tibia injury. Am J Pathol.

[38] Wang S, Xiao L, Prasadam I, Crawford R, Zhou Y, Xiao Y (2022). Inflammatory macrophages interrupt osteocyte maturation and mineralization via regulating the Notch signaling pathway. Mol Med.

[39] Duplomb L, Baud’huin M, Charrier C, Berreur M, Trichet V, Blanchard F (2008). Interleukin-6 inhibits receptor activator of nuclear factor kappaB ligand-induced osteoclastogenesis by diverting cells into the macrophage lineage: key role of Serine727 phosphorylation of signal transducer and activator of transcription 3. Endocrinology.

[40] Wolfe H, Minogue AM, Rooney S, Lynch MA (2018). Infiltrating macrophages contribute to age-related neuroinflammation in C57/BL6 mice. Mech Ageing Dev.

[41] Batoon L, Millard SM, Wullschleger ME, Preda C, Wu AC, Kaur S (2019). CD169(+) macrophages are critical for osteoblast maintenance and promote intramembranous and endochondral ossification during bone repair. Biomaterials.

[42] Mohamad SF, Xu L, Ghosh J, Childress PJ, Abeysekera I, Himes ER (2017). Osteomacs interact with megakaryocytes and osteoblasts to regulate murine hematopoietic stem cell function. Blood Adv.

[43] Chang MK, Raggatt LJ, Alexander KA, Kuliwaba JS, Fazzalari NL, Schroder K (2008). Osteal tissue macrophages are intercalated throughout human and mouse bone lining tissues and regulate osteoblast function in vitro and in vivo. J Immunol.

[44] Wu AC, He Y, Broomfield A, Paatan NJ, Harrington BS, Tseng HW (2016). CD169(+) macrophages mediate pathological formation of woven bone in skeletal lesions of prostate cancer. J Pathol.

[45] Batoon L, McCauley LK (2021). Cross talk between macrophages and cancer cells in the bone metastatic environment. Front Endocrinol (Lausanne).

[46] Batoon L, Koh AJ, Kannan R, McCauley LK, Roca H (2023). Caspase-9 driven murine model of selective cell apoptosis and efferocytosis. Cell Death Dis.

[47] Jilka RL, Weinstein RS, Bellido T, Parfitt AM, Manolagas SC (1998). Osteoblast programmed cell death (apoptosis): modulation by growth factors and cytokines. J Bone Miner Res.

[48] Metzger CE, Narayanan SA (2019). The role of osteocytes in inflammatory bone loss. Front Endocrinol (Lausanne).

[49] Batoon L, Millard SM, Raggatt LJ, Wu AC, Kaur S, Sun LWH (2021). Osteal macrophages support osteoclast-mediated resorption and contribute to bone pathology in a postmenopausal osteoporosis mouse model. J Bone Miner Res.

[50] Newby AC (2016). Metalloproteinase production from macrophages - a perfect storm leading to atherosclerotic plaque rupture and myocardial infarction. Exp Physiol.

[51] Huang WC, Sala-Newby GB, Susana A, Johnson JL, Newby AC (2012). Classical macrophage activation up-regulates several matrix metalloproteinases through mitogen activated protein kinases and nuclear factor-kappaB. PLoS One.

[52] Yoshida H, Hayashi S, Kunisada T, Ogawa M, Nishikawa S, Okamura H (1990). The murine mutation osteopetrosis is in the coding region of the macrophage colony stimulating factor gene. Nature.

[53] Wiktor-Jedrzejczak W, Bartocci A, Ferrante AW, Ahmed-Ansari A, Sell KW, Pollard JW (1990). Total absence of colony-stimulating factor 1 in the macrophage-deficient osteopetrotic (op/op) mouse. Proc Natl Acad Sci U S A.

[54] Takatsuka H, Umezu H, Hasegawa G, Usuda H, Ebe Y, Naito M (1998). Bone remodeling and macrophage differentiation in osteopetrosis (op) mutant mice defective in the production of macrophage colony-stimulating factor. J Submicrosc Cytol Pathol.

[55] Werner SL, Sharma R, Woodruff K, Horn D, Harris SE, Gorin Y (2020). CSF-1 in osteocytes inhibits Nox4-mediated oxidative stress and promotes normal bone homeostasis. JBMR Plus.

[56] Inoue K, Qin Y, Xia Y, Han J, Yuan R, Sun J, et al. Bone marrow Adipoq-lineage progenitors are a major cellular source of M-CSF that dominates bone marrow macrophage development, osteoclastogenesis, and bone mass. Elife. 2023;12. 10.7554/eLife.82118. 10.7554/eLife.82118 PMC10005769 36779851

[57] Zhong L, Yao L, Tower RJ, Wei Y, Miao Z, Park J, et al. Single cell transcriptomics identifies a unique adipose lineage cell population that regulates bone marrow environment. Elife. 2020;9. 10.7554/eLife.54695. 10.7554/eLife.54695 PMC7220380 32286228

[58] Takahashi N, Udagawa N, Kobayashi Y, Suda T (2007). Generation of osteoclasts in vitro, and assay of osteoclast activity. Methods Mol Med.

[59] Ambrosi TH, Marecic O, McArdle A, Sinha R, Gulati GS, Tong X (2021). Aged skeletal stem cells generate an inflammatory degenerative niche. Nature.

[60] Comazzetto S, Shen B, Morrison SJ (2021). Niches that regulate stem cells and hematopoiesis in adult bone marrow. Dev Cell.

[61] Winkler IG, Sims NA, Pettit AR, Barbier V, Nowlan B, Helwani F (2010). Bone marrow macrophages maintain hematopoietic stem cell (HSC) niches and their depletion mobilizes HSCs. Blood.

[62] Gekas C, Graf T (2013). CD41 expression marks myeloid-biased adult hematopoietic stem cells and increases with age. Blood.

[63] Rundberg Nilsson A, Soneji S, Adolfsson S, Bryder D, Pronk CJ (2016). Human and murine hematopoietic stem cell aging is associated with functional impairments and intrinsic megakaryocytic/erythroid bias. PLoS One.

[64] Frisch BJ, Hoffman CM, Latchney SE, LaMere MW, Myers J, Ashton J, et al. Aged marrow macrophages expand platelet-biased hematopoietic stem cells via interleukin1B. JCI Insight. 2019;5(10). 10.1172/jci.insight.124213. 10.1172/jci.insight.124213 PMC6542605 30998506

[65] Beaulieu LM, Lin E, Mick E, Koupenova M, Weinberg EO, Kramer CD (2014). Interleukin 1 receptor 1 and interleukin 1beta regulate megakaryocyte maturation, platelet activation, and transcript profile during inflammation in mice and humans. Arterioscler Thromb Vasc Biol.

[66] Haas S, Hansson J, Klimmeck D, Loeffler D, Velten L, Uckelmann H (2015). Inflammation-induced emergency megakaryopoiesis driven by hematopoietic stem cell-like megakaryocyte progenitors. Cell Stem Cell.

[67] Chitteti BR, Kobayashi M, Cheng Y, Zhang H, Poteat BA, Broxmeyer HE (2014). CD166 regulates human and murine hematopoietic stem cells and the hematopoietic niche. Blood.

[68] Khosla S, Farr JN, Tchkonia T, Kirkland JL (2020). The role of cellular senescence in ageing and endocrine disease. Nat Rev Endocrinol.

[69] Farr JN, Fraser DG, Wang H, Jaehn K, Ogrodnik MB, Weivoda MM (2016). Identification of senescent cells in the bone microenvironment. J Bone Miner Res.

[70] Farr JN, Xu M, Weivoda MM, Monroe DG, Fraser DG, Onken JL (2017). Targeting cellular senescence prevents age-related bone loss in mice. Nat Med.

[71] Coppe JP, Patil CK, Rodier F, Sun Y, Munoz DP, Goldstein J (2008). Senescence-associated secretory phenotypes reveal cell-nonautonomous functions of oncogenic RAS and the p53 tumor suppressor. PLoS Biol.

[72] Choi YW, Kim YH, Oh SY, Suh KW, Kim YS, Lee GY (2021). Senescent tumor cells build a cytokine shield in colorectal cancer. Adv Sci (Weinh).

[73] Saul D, Kosinsky RL, Atkinson EJ, Doolittle ML, Zhang X, LeBrasseur NK (2022). A new gene set identifies senescent cells and predicts senescence-associated pathways across tissues. Nat Commun.

[74] Schloesser D, Lindenthal L, Sauer J, Chung KJ, Chavakis T, Griesser E, et al. Senescent cells suppress macrophage-mediated corpse removal via upregulation of the CD47-QPCT/L axis. J Cell Biol. 2023;222(2). 10.1083/jcb.202207097. 10.1083/jcb.202207097 PMC9723804 36459066

[75] Sturmlechner I, Zhang C, Sine CC, van Deursen EJ, Jeganathan KB, Hamada N (2021). p21 produces a bioactive secretome that places stressed cells under immunosurveillance. Science.

[76] Kim YH, Choi YW, Lee J, Soh EY, Kim JH, Park TJ (2017). Senescent tumor cells lead the collective invasion in thyroid cancer. Nat Commun.

[77] Barkal AA, Weiskopf K, Kao KS, Gordon SR, Rosental B, Yiu YY (2018). Engagement of MHC class I by the inhibitory receptor LILRB1 suppresses macrophages and is a target of cancer immunotherapy. Nat Immunol.

[78] Prieto LI, Sturmlechner I, Graves SI, Zhang C, Goplen NP, Yi ES (2023). Senescent alveolar macrophages promote early-stage lung tumorigenesis. Cancer Cell.

[79] Haston S, Gonzalez-Gualda E, Morsli S, Ge J, Reen V, Calderwood A (2023). Clearance of senescent macrophages ameliorates tumorigenesis in KRAS-driven lung cancer. Cancer Cell.

[80] Li CJ, Xiao Y, Sun YC, He WZ, Liu L, Huang M (2021). Senescent immune cells release grancalcin to promote skeletal aging. Cell Metab.

[81] Hall BM, Balan V, Gleiberman AS, Strom E, Krasnov P, Virtuoso LP (2017). p16(Ink4a) and senescence-associated beta-galactosidase can be induced in macrophages as part of a reversible response to physiological stimuli. Aging (Albany NY).

[82] Behmoaras J, Gil J. Similarities and interplay between senescent cells and macrophages. J Cell Biol. 2021;220(2). 10.1083/jcb.202010162. 10.1083/jcb.202010162 PMC7769159 33355620

[83] Krasniewski LK, Chakraborty P, Cui CY, Mazan-Mamczarz K, Dunn C, Piao Y, et al. Single-cell analysis of skeletal muscle macrophages reveals age-associated functional subpopulations. Elife. 2022;11. 10.7554/eLife.77974. 10.7554/eLife.77974 PMC9629833 36259488

[84] Coulis G, Jaime D, Guerrero-Juarez C, Kastenschmidt JM, Farahat PK, Nguyen Q (2023). Single-cell and spatial transcriptomics identify a macrophage population associated with skeletal muscle fibrosis. Sci Adv.

[85] Millard SM, Heng O, Opperman KS, Sehgal A, Irvine KM, Kaur S (2021). Fragmentation of tissue-resident macrophages during isolation confounds analysis of single-cell preparations from mouse hematopoietic tissues. Cell Rep.

